# ACP-DRL: an anticancer peptides recognition method based on deep representation learning

**DOI:** 10.3389/fgene.2024.1376486

**Published:** 2024-04-09

**Authors:** Xiaofang Xu, Chaoran Li, Xinpu Yuan, Qiangjian Zhang, Yi Liu, Yunping Zhu, Tao Chen

**Affiliations:** ^1^ State Key Laboratory of Medical Proteomics, Beijing Proteome Research Center, National Center for Protein Sciences(Beijing), Beijing Institute of Lifeomics, Beijing, China; ^2^ Department of General Surgery, First Medical Center, Chinese PLA General Hospital, Beijing, China; ^3^ Institute of Dataspace, Hefei Comprehensive National Science Center, Hefei, China

**Keywords:** anticancer peptides, deep representation learning, BERT, self-supervised, pre-training, language models

## Abstract

Cancer, a significant global public health issue, resulted in about 10 million deaths in 2022. Anticancer peptides (ACPs), as a category of bioactive peptides, have emerged as a focal point in clinical cancer research due to their potential to inhibit tumor cell proliferation with minimal side effects. However, the recognition of ACPs through wet-lab experiments still faces challenges of low efficiency and high cost. Our work proposes a recognition method for ACPs named ACP-DRL based on deep representation learning, to address the challenges associated with the recognition of ACPs in wet-lab experiments. ACP-DRL marks initial exploration of integrating protein language models into ACPs recognition, employing in-domain further pre-training to enhance the development of deep representation learning. Simultaneously, it employs bidirectional long short-term memory networks to extract amino acid features from sequences. Consequently, ACP-DRL eliminates constraints on sequence length and the dependence on manual features, showcasing remarkable competitiveness in comparison with existing methods.

## 1 Introduction

Cancer is a major public health problem worldwide and one of the leading cause of death (Siegel et al., 2023). Typical treatment options to reduce the burden of cancer on human health involve surgery, radiotherapy, and/or systemic therapy. However, the toxicities associated with traditional treatment methods, present considerable challenges for tolerability and adherence, making it difficult for patients to complete their prescribed treatment regimens (Mun et al., 2018). Therefore, the development of new anticancer drugs with higher efficacy, low resistance, and fewer adverse effects are necessary. Anticancer peptides (ACPs) potentially offer new perspectives for achieving this goal (Gabernet et al., 2016). Considering their intrinsic nature as cationic amphiphiles, ACPs exhibit unique, receptor-independent mechanisms. These peptides display an exceptional capacity to selectively target and eliminate cancer cells via folding-dependent membrane disruption (Aronson et al., 2018). On the one hand, ACPs therapy has been extensively researched and applied in preclinical and various stages of clinical trials against tumors (Pelliccia et al., 2019; Liu et al., 2024). On the other hand, the time-consuming and costly process of identifying ACPs through biological experiments, as well as the limited number of available ACPs, have hindered its development.

Fortunately, with the tremendous progress made in the field of machine learning over the past decades, the feasibility of employing computational methods to predict typical peptides has become a reality. As a result, various recognition methods for ACPs based on amino acid sequences have emerged, such as iACP (Chen et al., 2016), PEPred-Suite (Wei et al., 2019), ACPred-Fuse (Rao et al., 2020), iACP-DRLF (Lv et al., 2021), AntiCP 2.0 (Agrawal et al., 2021), ACP-check (Zhu et al., 2022) and ACP-BC (Sun et al., 2023). These recognition methods adopt diverse approaches to convert amino acid sequences into numerical representations and use machine learning algorithms to uncover patterns within these features. Among these methodologies, AntiCP 2.0 relies on common feature extraction techniques such as dipeptide composition and an ETree classifier model. In contrast, iACP-DRLF leverages two deep representation learning techniques alongside LGBM for refined feature selection. And ACP-check integrates a bidirectional long short-term memory (Bi-LSTM) network with a fully connected network, facilitating predictions based on both raw amino acid sequences and handcrafted features. ACP-BC is a three-channel end-to-end model, which employs data augmentation techniques, integrated in various combinations.

Despite the numerous informatics approaches proposed for ACPs recognition, there is still room for improvement. For instance, AntiCP 2.0 imposes a requirement on the target peptide sequence length to be between 4 and 50, while iACP-DRLF introduces a complex feature extraction strategy. More importantly, the scarcity of experimentally annotated datasets of ACPs significantly constrains the utilization and performance of machine learning. In light of these considerations, this study proposes ACP-DRL. ACP-DRL incorporates advanced language models that can efficiently utilize vast unlabelled datasets and extend sequence length through positional encoding, while Bi-LSTM operates without imposing restrictions on sequence length. In ACP-DRL, we have shifted our focus to deep representation learning, alleviating the scarcity of ACP datasets through the application of extensive unlabeled data. This allows predictions on longer sequences and reduces dependence on feature engineering based on expert knowledge. Simultaneously, in comparison with existing methods, ACP-DRL demonstrates exceptional performance.

## 2 Materials and methods

### 2.1 Datasets

To ensure a fair comparison, the main and alternate datasets supplied by AntiCP 2.0 (Agrawal et al., 2021) were employed in this research. These consolidated datasets incorporate data harvested from numerous databases including DADP (Novković et al., 2012), CAMP (Waghu et al., 2014), APD (Wang and Wang, 2004), APD2 (Wang et al., 2009), CancerPPD (Tyagi et al., 2015), Uniprot (Consortium, 2015), and SwissProt (Gasteiger et al., 2001) databases. The positive dataset, enriched with experimentally validated ACPs, was derived from a conjoined compilation of the antimicrobial peptide (AMP) database and the CancerPPD database. In contrast, the main negative dataset consisted of AMPs lacking anticancer activity, sourced solely from the AMP database, while the alternative negative dataset encompassed random peptides extracted from protein within the SwissProt database (Gasteiger et al., 2001). The main dataset includes 861 ACPs and equal number of non-ACPs while the alternate dataset holds a count of 970 for both ACPs and non-ACPs.

We additionally created an imbalanced dataset (comprising 845 ACPs and 3,800 non-ACPs) for five-fold cross-validation, which encompasses all data from both the main and alternate datasets. Additional sequence data was obtained from Rao et al. (2020), and we used the CD-HIT algorithm to construct nonredundant sequences.

Furthermore, we collected approximately 1.5 million peptide sequences from PeptideAtlas (Omenn et al., 2022) as an unlabeled dataset for in-domain further pre-training of protein language model.

We assessed the amino acid composition (AAC) of peptides and generated six sample sequence logos (Supplementary Figure S1) in the in-domain further pre-training daset (IFPT), main, and alternate datasets. This was done to gain insights into the residue preferences at the N-terminus and C-terminus in these three datasets.

The result indicates that both the main and alternate datasets showed a high predominance of ‘K’, ‘L’, and ‘A’ residues at the N-terminus, and ‘K’ and ‘L’ at the C-terminus (Supplementary Figure S1A), consistent with previous studies (Agrawal et al., 2021). However, no particular amino acid type dominated at the N-terminus (Supplementary Figure S1A) in the IFPT dataset, suggesting little to no conservation. As for the C-terminus (Supplementary Figure S1B), it often concluded with either ‘K’ or ‘R’, most likely influenced by specific enzyme cleavage sites, as the C-terminus is the end part to form during protein synthesis. The presence of amino acids such as lysine or arginine could have a significant impact on this cleavage process, with enzymes like trypsin specifically cleaving these, thereby affecting their prevalence at the C-termini. It can be discerned that the dataset used for in-domain further pre-training does not exhibit substantial similarity with the dataset utilized for anticancer peptide recognition.

### 2.2 Framework of ACP-DRL

**FIGURE 1:**
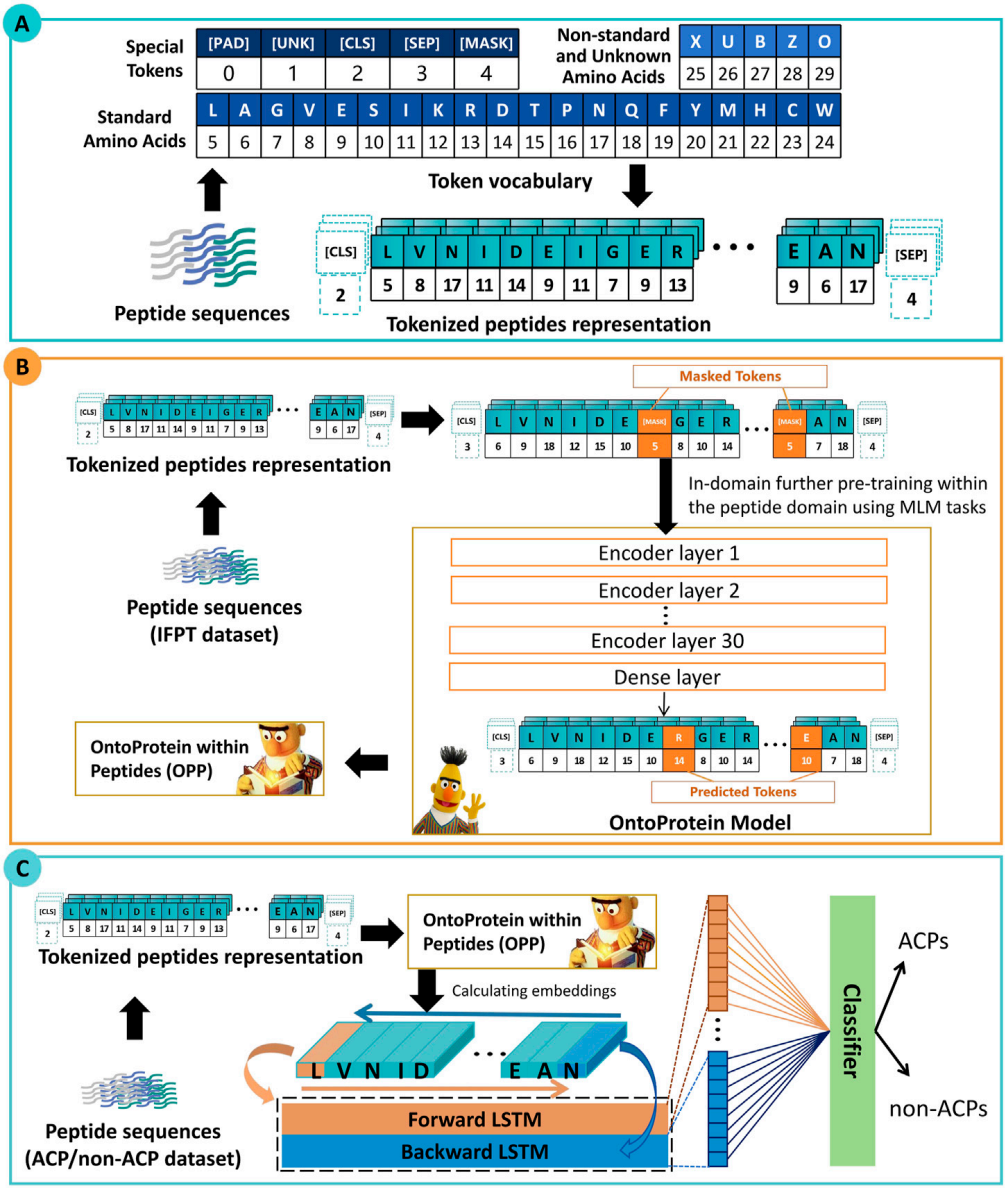
Framework of ACP-DRL. **(A)** Tokenized peptides representation. **(B)** Language model with in-domain further pre-training. **(C)** Fine-tuning layer and classifier.

As depicted in Figure 1, the framework of ACP-DRL consists of three main modules. Firstly, the initial section delineates the representation of peptide sequences. Secondly, the following section elucidates the further pre-training of the protein language model. Thirdly, the section explains the process of extracting peptide sequence features using a Bi-LSTM, and subsequently classifying these peptides based on the extracted features.

#### 2.2.1 Tokenized peptides representation

The initial section of Figure 1 illustrates a process in which peptide sequences are tokenized, which means that each amino acid is converted into its corresponding numerical IDs. These IDs are subsequently used as inputs for our peptide language model. Within the vocabulary of our language model, a total of 26 tokens have been utilized. This includes five special tokens ([PAD] [UNK] [CLS] [SEP] [MASK]), 20 tokens representing the standard amino acids in their abbreviated forms. Additionally, the token “X” has been specifically designated to denote non-generic or unresolved amino acids. This allocation of “X” facilitates the accommodation of non-standard amino acids, ultimately enhancing the model’s adaptability and flexibility.

#### 2.2.2 Language model with in-domain further pre-training

There is a perspective within the community that proteins can be represented by amino acids, and thus, they can be approximated as a unique form of natural language (Ofer et al., 2021). Recent academic research has further emphasized this perspective, with the release of numerous protein language models. Elnaggar et al. (2021) put forward the BERT-BFD model which was trained on the BFD (Steinegger et al., 2019) dataset composed of an impressive count of 2,122 million protein sequences. Concurrently, OntoProtein was put forth by Zhang et al. (2022), employing the robust techniques of knowledge graphs and gene ontology. The aforementioned efforts have yielded excellent protein language models. Upon consideration, we selected OntoProtein as the foundational model and further conducted training based on our work.

Pre-training broadly involves the initial training of a model on a large dataset which enables it to acquire universal features. In-domain further pre-training signifies an added layer of refinement to the pre-trained model using task-relevant data within a specific field or operation. This additional step aims to bolster model performance within its designated tasks (Grambow et al., 2022).

In the context of our research, we collected and employed the IFPT dataset (about 1.5 million peptide sequences) to incrementally enhance OntoProtein to approximate the peptide level feature space more closely. Through this strategy, we proposed the OntoProtein within Peptides (OPP) model and could continuously obtain and train learnable deep representations during the training of downstream tasks.

The imperative behind this step is to facilitate OntoProtein’s adaptability to the transition happening from protein sequences to peptide sequences. It is worth noting that, although OntoProtein jointly trains knowledge embedding (KE) and masked language modeling (MLM) tasks, during the In-domain further pre-training stage, we only trained the MLM task.

As shown in Figure 1, during the in-domain further pre-training stage, a subset of amino acids is masked, and the language model needs to predict the masked amino acids based on contextual information. This prompts the language model to learn the underlying information of peptide sequences. In our approach, each token (amino acid) has a 15% probability of being masked, and we use cross-entropy loss to estimate the predictions for these masked tokens. This process invokes the preparation of masked token inputs, conforming to the principles of masked language modeling. The distribution of these masked tokens adheres to a specific ratio: 80% are masked, 10% are replaced with random tokens, and the remaining 10% maintain their original identity.

#### 2.2.3 Fine-tuning layer and classifier

BERT has demonstrated significant potential in the field of text classification, with researchers commonly acknowledging that the “[CLS]” token is expected to capture information from the entire sequence (Sun et al., 2019; Wang and Kuo, 2020). Consequently, in early classification tasks, researchers often relied solely on the information from the “[CLS]” token; however, this practice is not considered optimal (Jiang et al., 2021; Kim et al., 2021). In order to further extract sequence features from the peptides, we added an extra fine-tuning layer rather than connecting the “[CLS]” output directly to a fully connected layer. Bi-LSTM is particularly suitable for handling sequence data and can simultaneously capture both preceding and following contextual information.

The LSTM comprises four components: the forgetting gate *f*_*t*_, the input gate *i*_*t*_, the cell state *C*_*t*_, and the output gate *o*_*t*_. The forgetting gate *f*_*t*_ takes a value between 0 and 1. When an element of *f*_*t*_ is 0, it prevents the passage of the value from the previous cell state *C*_*t*−1_, achieving selective forgetfulness. Meanwhile, the input gate *i*_*t*_ contributes information to the cell state *C*_*t*_, thereby updating the information. This selective interplay of remembering and forgetting effectively addresses challenges such as gradient explosion, gradient disappearance, and distance-dependent issues commonly encountered in traditional RNNs. The whole process is as follows:

$$f_{t}=\sigma \left(W_{f}\cdot \left[h_{t-1},X_{t}\right]+b_{f}\right)$$ (1)

$$i_{t}=\sigma \left(W_{i}\cdot \left[h_{t-1},X_{t}\right]+b_{i}\right)$$ (2)

$${\tilde{C}}_{t}=\tanh\left(W_{C}\cdot \left[h_{t-1},X_{t}\right]+b_{C}\right)$$ (3)

$$C_{t}=C_{t-1}◦f_{t}+i_{t}◦{\tilde{C}}_{t}$$ (4)

$$o_{t}=\sigma \left(W_{o}\cdot \left[h_{t-1},X_{t}\right]+b_{o}\right)$$ (5)

$$h_{t}=o_{t}◦\tanh\left[C_{t}\right]$$ (6)

In this study, we employed Bi-LSTM to extract contextual information. As illustrated in the third section of Figure 1, our OPP model furnishes a high-dimensional encoding for each amino acid in peptide sequences. We sequentially input this into two LSTMs (forward and backward) and combined their state vectors to provide a feature vector for each peptide. After that, we utilize a fully connected layer and Softmax function for classification, with a default threshold of 0.5. If the probability of belonging to the positive class is greater than 0.5, the target peptide sequence is categorized as an ACP; otherwise, it is designated as a non-ACP.

### 2.3 Performance evaluation

The evaluation in this study is conducted using four metrics, namely, accuracy (Acc), sensitivity (Sen), specificity (SP) and Mathew’s correlation coefficient (MCC), which is in line with previous studies. The specific evaluation metrics are as follows:

$$Acc=\frac{TP+TN}{TP+TN+FP+FN}$$ (7)

$$Sen=\frac{TP}{TP+FN}$$ (8)

$$Spc=\frac{TN}{TN+FP}$$ (9)

$$MCC=\frac{TP\times TN-FP\times FN}{\sqrt{\left(TP+FN\right)\times \left(TP+FP\right)\times \left(TN+FP\right)\times \left(TN+FN\right)}}$$ (10)

## 3 Results and discussion

We ran ACP-DRL on a single node of the GPU cluster in the National Center for Protein Sciences (Beijing). During the training, we trained for 20 epochs with a learning rate of 2e-5 on 8 T V100 GPUs, using adafactor as the optimizer, and adopted the cosine with restarts learning rate schedule. The batch size could be set to 32 for each training iteration. In this section, we commence with the evaluation of the language model, followed by an assessment of various fine-tuning layers. Finally, we compare results of ACP-DRL with existing methods.

### 3.1 Evaluation of different language models

**FIGURE 2:**
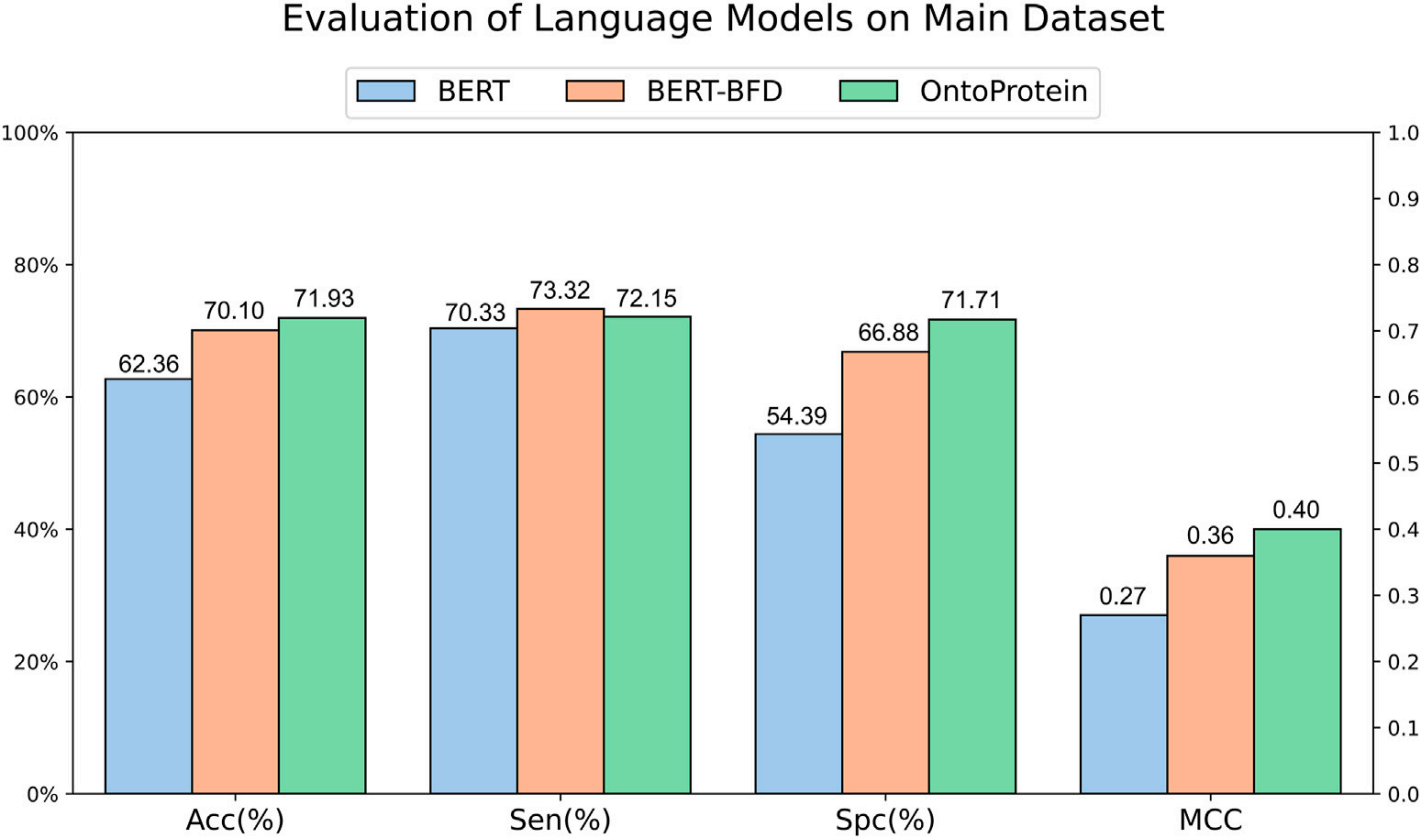
Evaluation of language models on main dataset.

The development of Artificial Intelligence for Science has provided scholars with available protein language models. To assess the feasibility of current typical protein language models in ACPs recognition, we gathered randomly initialized BERT, BERT-BFD trained on 2,122 million protein sequences and OntoProtein which incorporates joint training with KE and GO, for training and evaluation. We designed a common vocabulary for these three models, encoding peptide sequences from the main dataset into each model, and subsequently employing a fully connected layer for classification on the encoded results. The evaluation results (Figure 2) suggest that the three language models have similar performances in terms of sensitivity. Still, regarding specificity, the initialized BERT performs worse, which might contribute to its lower accuracy. BERT-BFD and OntoProtein, both of which have been pre-trained employing a substantial volume of protein sequences, demonstrate performances that are relatively equivalent. Overall, OntoProtein is slightly inferior to BERT-BFD in sensitivity but achieves advantages in accuracy, specificity and MCC, with the benefit in specificity being more pronounced. Furthermore, considering its slightly higher MCC than BERT-BFD, we propose that OntoProtein has more potential for the task at hand.

### 3.2 Performance of different fine-tuning layers

**FIGURE 3:**
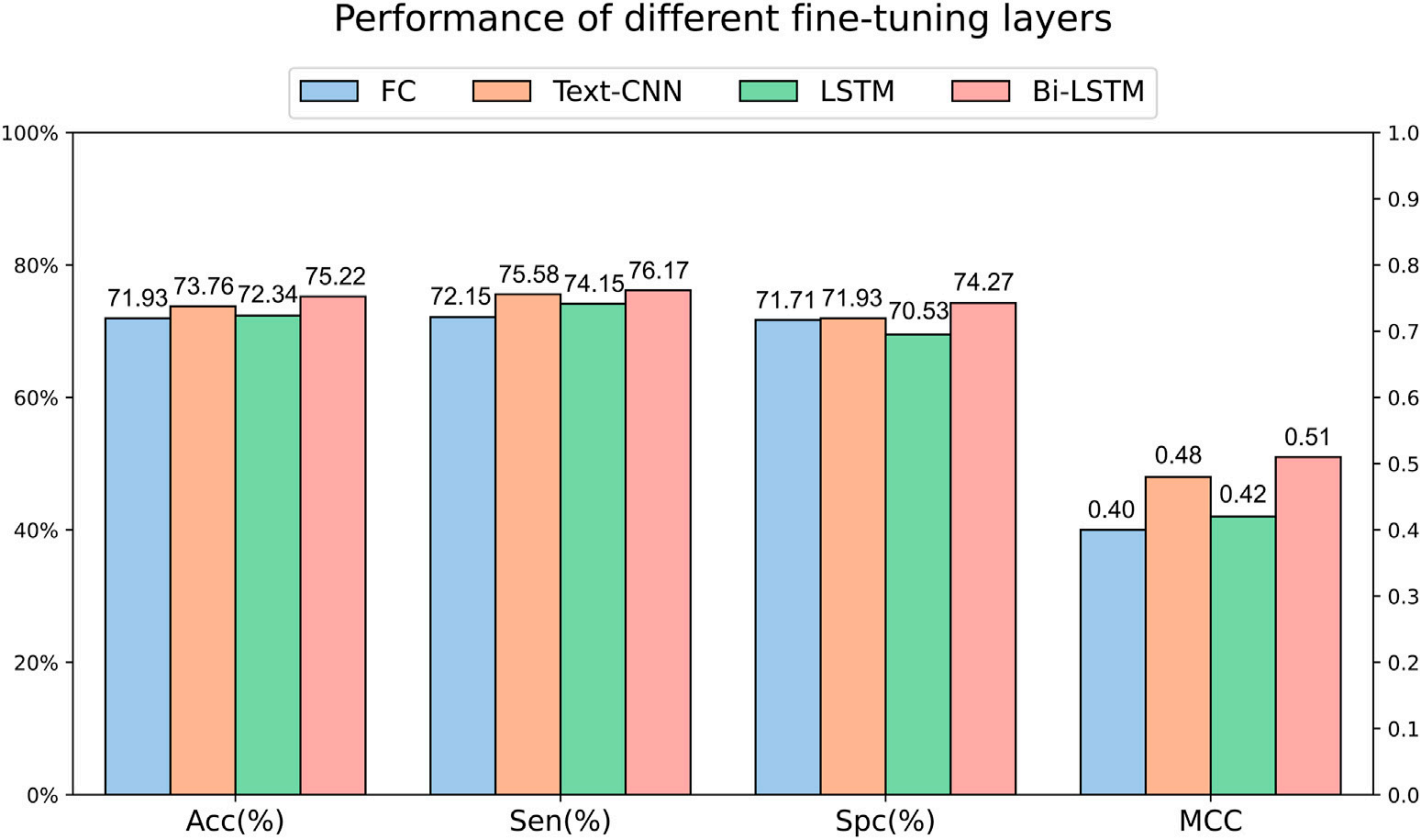
Performance of different fine-tuning layers on main dataset.

We can obtain the encoding of each amino acid in a peptide sequence through language models. To further extract sequence features, we utilized OntoProtein as the base model and experimented with various fine-tuning layers on the main dataset. Specifically, the fully connected layer only utilized the encoding of the “[CLS]” token for classification, while Text-CNN, forward LSTM, and Bi-LSTM utilized the encoding information of the entire sequence. Figure 3 illustrates the experimental results under different fine-tuning layers. It can be observed that the effectiveness of using only the encoding of the “[CLS]” token for classification is not satisfactory, corroborating the findings of Kim et al. (2021) and Jiang et al. (2021). The performance is somewhat improved with a simple forward LSTM, but a significant leap is observed when incorporating a backward LSTM. Text-CNN demonstrates a certain level of competitiveness in this task but falls short of Bi-LSTM, reaffirming our confidence in choosing Bi-LSTM as the fine-tuning layer.

### 3.3 Evaluation of in-domain further pre-training

**FIGURE 4:**
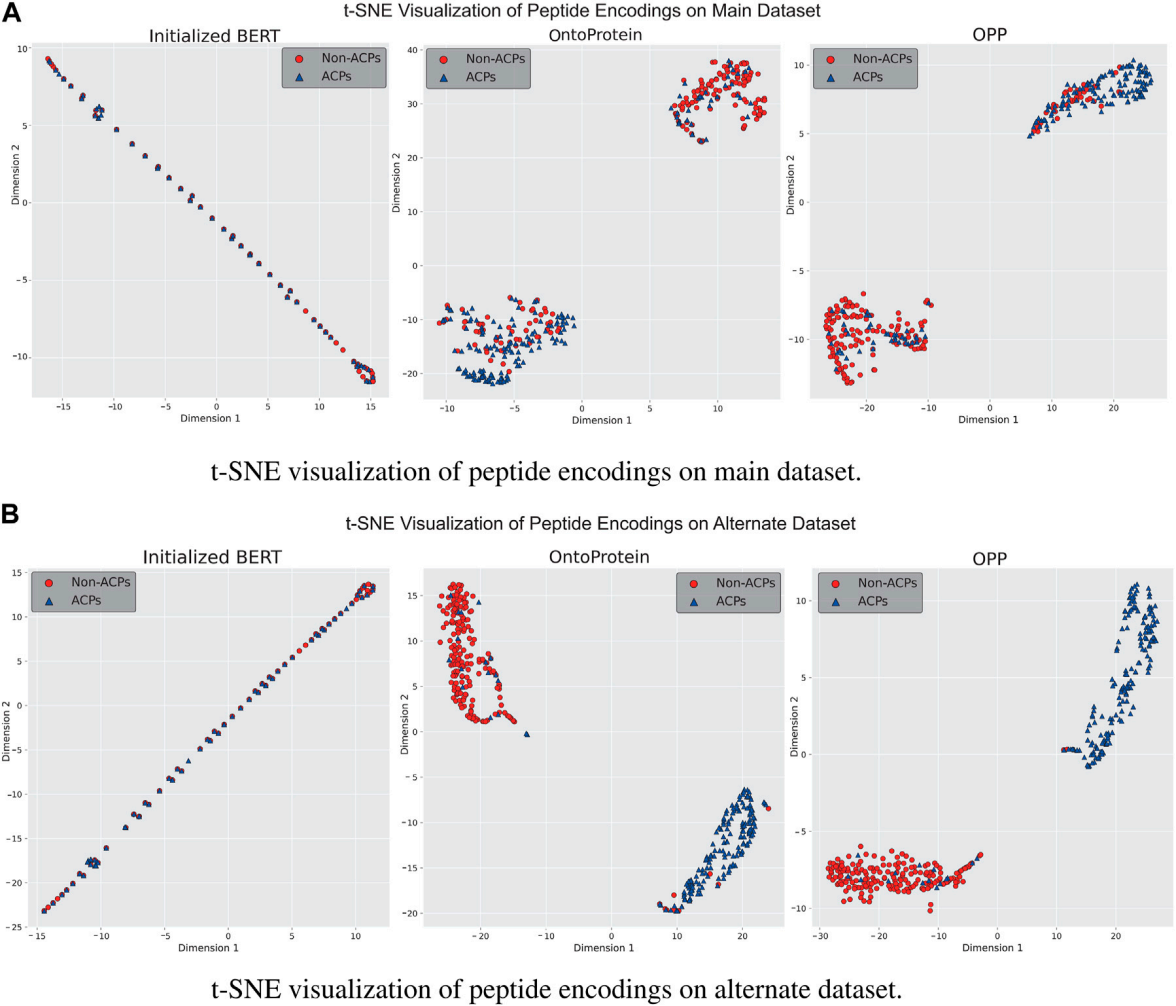
Visualization results of t-SNE for language models at different stages on **(A)** main dataset and **(B)** alternate dataset.

In-domain further pre-training is a primary approach for enhancing language models using in-domain additional datasets. We gathered a dataset comprising approximately 1.5 million peptide sequences to assess performance of OntoProtein in the peptide domain, ultimately obtaining the OPP model used for actual training. To better understand the distribution changes of feature information in language models, we employed t-Distributed Stochastic Neighbor Embedding (t-SNE) for the visualization of model features. We discussed three stages of the language model: a) the unpretrained BERT model, b) the OntoProtein model released by Zhang et al. (2022), and c) the OPP model obtained through our additional pre-training. Figures 4A, B present the t-SNE visualization results of the test sets from the main and alternate datasets at three stages. It can be observed that the initialized BERT exhibits a considerable overlap of points on both datasets, confirming the subpar testing results shown in Figure 2. This phenomenon may be attributed to the model’s excessive parameter count compared to the small training dataset. OntoProtein and our OPP model demonstrate excellent performance in the t-SNE visualizations, displaying distinct sample clusters. On the main dataset, the ACPs of our OPP model are more clustered than those of OntoProtein, while on the alternate dataset, the OPP model has fewer mixed-in non-ACPs among its ACPs. Therefore, it is reasonable to conclude that the in-domain further pre-training strategy—utilizing the IFPT dataset implemented in this study—augments the model’s performance on both the main and alternate datasets. This enhancement is observable notwithstanding the significant differences in amino acid composition and positional preference between the unlabeled IFPT dataset and the datasets used for downstream tasks, as depicted in Supplementary Figure S1.

### 3.4 Comparison with existing methods

#### 3.4.1 Evaluation on the main and alternate dataset

After confirming the superior performance of our OPP model, we adopted it to construct the ACP-DRL model. To evaluate the performance of ACP-DRL model, we conduct a comparison with other machine learning or deep learning models, which include iACP, PEPred-Suite, ACPred-Fuse, AntiCP 2.0, iACP-DRLF, ACP-check, and ACP-BC. In this evaluation, we use the benchmark datasets (main and alternate datasets) as proposed by AntiCP 2.0.

The original ACP-BC paper does not furnish performance metrics for our benchmark datasets. Hence, using their GitHub repository (https://github.com/shunmengfan/ACP-BC) where their source code is available, we conducted experiments on our benchmark dataset using the best parameters stated in their paper. The optimal parameters deployed were: data augmentation factor R) set to 1.0, LSTM hidden layer C) with 256 nodes, number of neurons in the embedding layer D) as 512, and a learning rate of 1e-3. Both ACP-check and iACP-DRLF adopted the metric values reported in their respective papers, and hence there are slight differences in the degree of precision. The precision of ACP-check is maintained at 1%, while iACP-DRLF maintains its precision to 0.1%. The performance of the remaining methods came from the metric values reported by AntiCP 2.0 after executing evaluations on the main and alternate datasets.

Table 1 and Table 2 respectively display the performance on the main and alternate datasets, with the best performance for each metric highlighted in bold. As shown in Table 1, our model achieved the highest accuracy, specificity, and MCC on the main dataset, with a sensitivity close to that of ACP-check. The advantage is even more pronounced on the alternate dataset (Table 2), where our model reached an accuracy of 94.43%. Although our sensitivity was slightly lower than ACP-check, our specificity exceeded ACP-check by 3.64%. Overall, compared to existing advanced methods, the ACP-DRL model proposed in this study is highly competitive.

**TABLE 1:** Comparison with existing methods on main dataset.

	Acc(%)	Sen(%)	Spc (%)	MCC
ACP-DRL (Ours)	**78.96**	79.53	**78.39**	**0.56**
ACP-BC	75.16	72.61	77.71	0.50
ACP-check	78	**80**	77	0.56
iACP-DRLF	77.5	80.7	74.3	0.55
AntiCP 2.0	75.43	77.46	73.41	0.51
ACPred-Fuse	68.9	69.19	68.6	0.38
PEPred-Suite	53.49	33.14	73.84	0.08
iACP	55.10	77.91	32.16	0.11

The best performance for each metric highlighted in bold.

**TABLE 2:** Comparison with existing methods on alternate dataset.

	Acc(%)	Sen(%)	Spc (%)	MCC
ACP-DRL (Ours)	**94.43**	92.22	**96.64**	**0.89**
ACP-BC	91.05	92.14	89.96	0.82
ACP-check	93	**93**	93	0.86
iACP-DRLF	93.0	89.6	96.4	0.86
AntiCP 2.0	92.01	92.27	91.75	0.84
ACPred-Fuse	78.87	64.43	93.3	0.6
PEPred-Suite	57.47	40.21	74.74	0.16
iACP	77.58	78.35	76.8	0.55

The best performance for each metric highlighted in bold.

#### 3.4.2 Five-fold cross-validation on imbalanced dataset

To further illustrate the effectiveness of our model, we constructed an imbalanced dataset (comprising 845 ACPs and 3,800 non-ACPs) for a five-fold cross-validation. For this validation, we chose ACP-BC, the most recent model, and ACP-check, which offers competitive performance on main and alternate datasets, for comparisons.

We downloaded the source code for ACP-check from an open-source project (https://github.com/ystillen/ACP-check) and tailored a version for our five-fold cross-validation. For ACP-check, we chose the parameters best suited to the main dataset (lr = 1e-3, batch size = 50, epoch = 30). For ACP-BC, we still referred to the previously mentioned code and optimal parameters.

In this cross-validation, we included two additional evaluation metrics—Area Under the ROC Curve (AUC) and Area Under the Precision-Recall Curve (AUPR)—to further assess the model. While AUC serves as a common indicator for classifying performance across different thresholds (with a score close to 1.0 indicating strong performance), AUPR focuses more on the performance of classifiers in circumstances with imbalanced positive and negative samples.

Supplementary Tables S1–S3 demonstrate the performance of the three models on the imbalanced dataset, while Table 3 presents the average performance based on five-fold cross-validation, with the best results highlighted in bold. The results suggest that ACP-DRL has achieved top-tier performance across five evaluation metrics—Acc, Spc, MCC, AUC, and AUPR.

**TABLE 3:** Comparison of ACP-BC, ACP-check, and ACP-DRL in five-fold cross validation on an imbalanced dataset.

	Acc(%)	Sen(%)	Spc (%)	MCC	AUC	AUPR
ACP-BC	88.53	**64.58**	93.87	0.60	0.89	0.71
ACP-check	82.00	49.22	89.21	0.40	0.76	0.44
ACP-DRL (ours)	**89.82**	62.47	**95.89**	**0.64**	**0.91**	**0.78**

The best performance for each metric highlighted in bold.

Although ACP-BC attained the highest score for sensitivity, ACP-DRL achieved similar results whilst surpassing ACP-BC in specificity. This may suggest that ACP-DRL adopts a more conservative approach when classifying positive instances, hence avoiding potential misidentifications of true positives. Perhaps due to the sensitivity of ACP-check to data distribution, it did not demonstrate competitive performance in this test.

Paired T-tests were conducted on the results of five-fold cross-validation (as shown in Supplementary Table S4). The results indicated statistically significant differences in Spc, AUC, and AUPR metrics (*p* < 0.05) and a marginal difference in Acc (*p* = 0.05) when comparing our ACP-DRL model with ACP-BC. Meanwhile, when comparing with ACP-check, Acc, Sen, MCC, AUC, and AUPR all manifested significant differences (*p* < 0.05).

## 4 Conclusion

In this work, we have proposed a novel ACPs recognition method called ACP-DRL. ACP-DRL enhances the existing protein language model using in-domain further pre-training technology to approximate the peptide level feature space more closely, continuously obtains and trains learnable deep representation during training of downstream tasks, and learns the features at the amino acid level through Bi-LSTM, which combined with a fully connected layer to complete the recognition of ACPs. This design introduces the BERT-based protein large language model and further pre-training techniques into the ACPs recognition for the first time, eliminates constraints on sequence length and the dependence on manual features, showcasing remarkable competitiveness in comparison with existing methods. In recent years, recognizing various functional peptides like MFTP (Fan et al., 2023), MLBP(Tang et al., 2022), and PrMFTP (Yan et al., 2022) has seen significant advancements. These methods universally use encoders to transition peptide sequences into vectors. Believing that our OPP model is notably adept at this encoding task, we plan to apply it to the research in recognizing multifunctional peptides next.

## Data Availability

Publicly available datasets were analyzed in this study. Code and datasets can be found here: https://github.com/shallFun4Learning/ACP-DRL.

## References

[B1] Agrawal P. Bhagat D. Mahalwal M. Sharma N. Raghava G. P. (2021). Anticp 2.0: an updated model for predicting anticancer peptides. Briefings Bioinforma. 22, bbaa153. 10.1093/bib/bbaa153 32770192

[B2] Aronson M. R. Simonson A. W. Orchard L. M. Llinás M. Medina S. H. (2018). Lipopeptisomes: anticancer peptide-assembled particles for fusolytic oncotherapy. Acta Biomater. 80, 269–277. 10.1016/j.actbio.2018.09.025 30240951

[B3] Chen W. Ding H. Feng P. Lin H. Chou K.-C. (2016). iacp: a sequence-based tool for identifying anticancer peptides. Oncotarget 7, 16895–16909. 10.18632/oncotarget.7815 26942877 PMC4941358

[B4] Consortium U. (2015). Uniprot: a hub for protein information. Nucleic acids Res. 43, D204–D212. 10.1093/nar/gku989 25348405 PMC4384041

[B5] Elnaggar A. Heinzinger M. Dallago C. Rehawi G. Yu W. Jones L. (2021). Prottrans: towards cracking the language of lifes code through self-supervised deep learning and high performance computing. IEEE Trans. Pattern Analysis Mach. Intell. 43, 1–16. 10.1109/TPAMI.2019.2929146

[B6] Fan H. Yan W. Wang L. Liu J. Bin Y. Xia J. (2023). Deep learning-based multi-functional therapeutic peptides prediction with a multi-label focal dice loss function. Bioinformatics 39, btad334. 10.1093/bioinformatics/btad334 37216900 PMC10234765

[B7] Gabernet G. Müller A. T. Hiss J. A. Schneider G. (2016). Membranolytic anticancer peptides. MedChemComm 7, 2232–2245. 10.1039/c6md00376a

[B8] Gasteiger E. Jung E. Bairoch A. (2001). Swiss-prot: connecting biomolecular knowledge via a protein database. Curr. issues Mol. Biol. 3, 47–55. 10.21775/cimb.003.047 11488411

[B9] Grambow C. Zhang L. Schaaf T. (2022). In-domain pre-training improves clinical note generation from doctor-patient conversations. Proc. First Workshop Nat. Lang. Generation Healthc., 9–22.

[B10] Jiang Z. Tang R. Xin J. Lin J. (2021). How does bert rerank passages? an attribution analysis with information bottlenecks. Proc. Fourth BlackboxNLP Workshop Anal. Interpreting Neural Netw. NLP, 496–509. 10.18653/v1/2021.blackboxnlp-1.39

[B11] Kim T. Yoo K. M. Lee S.-g. (2021). “Self-guided contrastive learning for bert sentence representations,” in Proceedings of the 59th Annual Meeting of the Association for Computational Linguistics and the 11th International Joint Conference on Natural Language Processing (Volume 1: Long Papers), August 1-6, 2021, 2528–2540.

[B12] Liu H. Shen W. Liu W. Yang Z. Yin D. Xiao C. (2024). From oncolytic peptides to oncolytic polymers: a new paradigm for oncotherapy. Bioact. Mater. 31, 206–230. 10.1016/j.bioactmat.2023.08.007 37637082 PMC10450358

[B13] Lv Z. Cui F. Zou Q. Zhang L. Xu L. (2021). Anticancer peptides prediction with deep representation learning features. Briefings Bioinforma. 22, bbab008. 10.1093/bib/bbab008 33529337

[B14] Mun E. J. Babiker H. M. Weinberg U. Kirson E. D. Von Hoff D. D. (2018). Tumor-treating fields: a fourth modality in cancer treatment. Clin. Cancer Res. 24, 266–275. 10.1158/1078-0432.CCR-17-1117 28765323

[B15] Novković M. Simunić J. Bojović V. Tossi A. Juretić D. (2012). Dadp: the database of anuran defense peptides. Bioinformatics 28, 1406–1407. 10.1093/bioinformatics/bts141 22467909

[B16] Ofer D. Brandes N. Linial M. (2021). The language of proteins: NLP, machine learning and protein sequences. Comput. Struct. Biotechnol. J. 19, 1750–1758. 10.1016/j.csbj.2021.03.022 33897979 PMC8050421

[B17] Omenn G. S. Lane L. Overall C. M. Pineau C. Packer N. H. Cristea I. M. (2022). The 2022 report on the human proteome from the hupo human proteome project. J. proteome Res. 22, 1024–1042. 10.1021/acs.jproteome.2c00498 36318223 PMC10081950

[B18] Pelliccia S. Amato J. Capasso D. Di Gaetano S. Massarotti A. Piccolo M. (2019). Bio-inspired dual-selective bcl-2/c-myc g-quadruplex binders: design, synthesis, and anticancer activity of drug-like imidazo [2, 1-i] purine derivatives. J. Med. Chem. 63, 2035–2050. 10.1021/acs.jmedchem.9b00262 31241946

[B19] Rao B. Zhou C. Zhang G. Su R. Wei L. (2020). Acpred-fuse: fusing multi-view information improves the prediction of anticancer peptides. Briefings Bioinforma. 21, 1846–1855. 10.1093/bib/bbz088 31729528

[B20] Siegel R. L. Miller K. D. Wagle N. S. Jemal A. (2023). Cancer statistics, 2023. Ca Cancer J. Clin. 73, 17–48. 10.3322/caac.21763 36633525

[B21] Steinegger M. Mirdita M. Söding J. (2019). Protein-level assembly increases protein sequence recovery from metagenomic samples manyfold. Nat. methods 16, 603–606. 10.1038/s41592-019-0437-4 31235882

[B22] Sun M. Hu H. Pang W. Zhou Y. (2023). Acp-bc: a model for accurate identification of anticancer peptides based on fusion features of bidirectional long short-term memory and chemically derived information. Int. J. Mol. Sci. 24, 15447. 10.3390/ijms242015447 37895128 PMC10607064

[B23] Sun S. Cheng Y. Gan Z. Liu J. (2019). “Patient knowledge distillation for bert model compression,” in Proceedings of the 2019 Conference on Empirical Methods in Natural Language Processing and the 9th International Joint Conference on Natural Language Processing.

[B24] Tang W. Dai R. Yan W. Zhang W. Bin Y. Xia E. (2022). Identifying multi-functional bioactive peptide functions using multi-label deep learning. Briefings Bioinforma. 23, bbab414. 10.1093/bib/bbab414 34651655

[B25] Tyagi A. Tuknait A. Anand P. Gupta S. Sharma M. Mathur D. (2015). Cancerppd: a database of anticancer peptides and proteins. Nucleic acids Res. 43, D837–D843. 10.1093/nar/gku892 25270878 PMC4384006

[B26] Waghu F. H. Gopi L. Barai R. S. Ramteke P. Nizami B. Idicula-Thomas S. (2014). Camp: collection of sequences and structures of antimicrobial peptides. Nucleic acids Res. 42, D1154–D1158. 10.1093/nar/gkt1157 24265220 PMC3964954

[B27] Wang B. Kuo C.-C. J. (2020). Sbert-wk: a sentence embedding method by dissecting bert-based word models. IEEE/ACM Trans. Audio, Speech, Lang. Process. 28, 2146–2157. 10.1109/taslp.2020.3008390

[B28] Wang G. Li X. Wang Z. (2009). Apd2: the updated antimicrobial peptide database and its application in peptide design. Nucleic acids Res. 37, D933–D937. 10.1093/nar/gkn823 18957441 PMC2686604

[B29] Wang Z. Wang G. (2004). Apd: the antimicrobial peptide database. Nucleic acids Res. 32, D590–D592. 10.1093/nar/gkh025 14681488 PMC308759

[B30] Wei L. Zhou C. Su R. Zou Q. (2019). Pepred-suite: improved and robust prediction of therapeutic peptides using adaptive feature representation learning. Bioinformatics 35, 4272–4280. 10.1093/bioinformatics/btz246 30994882

[B31] Yan W. Tang W. Wang L. Bin Y. Xia J. (2022). Prmftp: multi-functional therapeutic peptides prediction based on multi-head self-attention mechanism and class weight optimization. PLoS Comput. Biol. 18, e1010511. 10.1371/journal.pcbi.1010511 36094961 PMC9499272

[B32] Zhang N. Bi Z. Liang X. Cheng S. Hong H. Deng S. (2022). “Ontoprotein: protein pretraining with gene ontology embedding,” in International Conference on Learning Representations, May 3-7, 2021.

[B33] Zhu L. Ye C. Hu X. Yang S. Zhu C. (2022). Acp-check: an anticancer peptide prediction model based on bidirectional long short-term memory and multi-features fusion strategy. Comput. Biol. Med. 148, 105868. 10.1016/j.compbiomed.2022.105868 35868046

