# Supplementary material for: ACP-DRL: an anticancer peptides recognition method based on deep representation learning
Source: Front Genet. 2024 Apr 9;15:1376486. doi: 10.3389/fgene.2024.1376486 (PMC11035771; doi:10.3389/fgene.2024.1376486)
Supplement: Supplementary file 1 [file DataSheet1.PDF]

frontiers

Supplementary Material

1 SUPPLEMENTARY TABLES AND FIGURES

Table S1. Five-fold cross validation of ACP-DRL on imbalanced datasets

	Acc(%)	Sen(%)	Spc(%)	MCC	AUC	AUPR
1	90.10	61.15	95.98	0.62	0.92	0.78
2	90.42	64.46	96.07	0.65	0.91	0.77
3	89.56	64.71	95.13	0.63	0.91	0.78
4	89.67	58.14	96.83	0.63	0.92	0.79
5	89.34	63.89	95.46	0.64	0.89	0.78

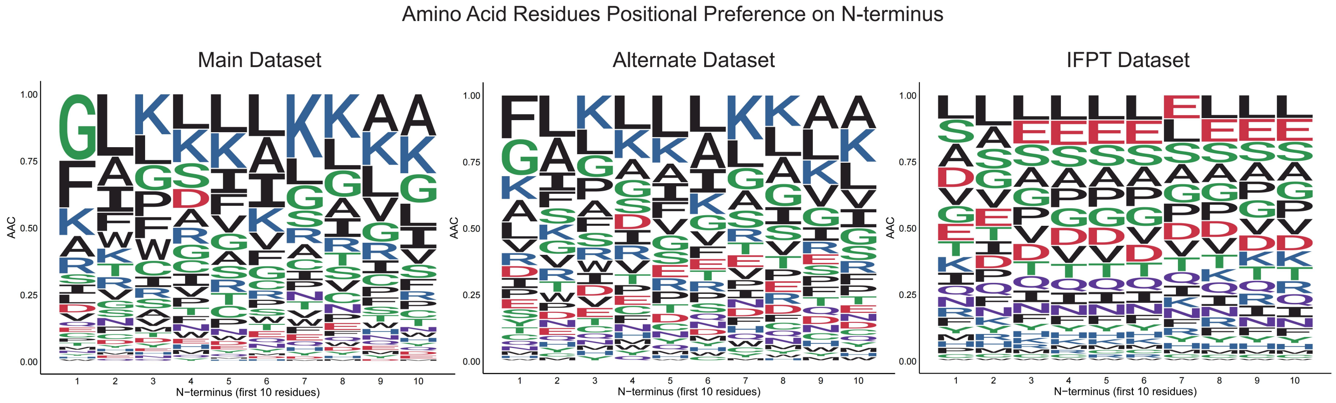

Figure 1a. Amino acid residues positional preference on N-terminus.

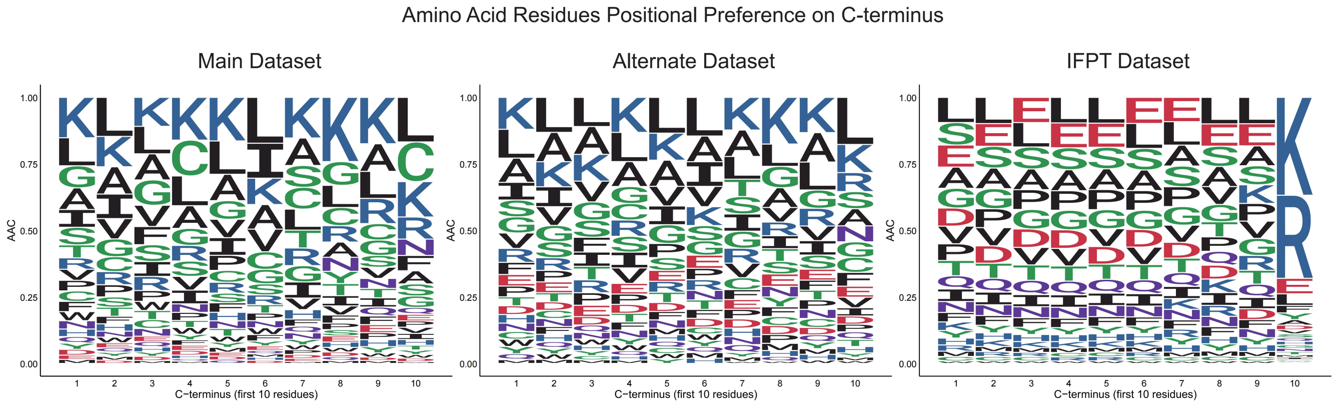

Figure 1b. Amino acid residues positional preference on C-terminus.

Figure 1. Sequence logos generated from N-terminus (first 10 residues) and C-terminus (last 10 residues) of peptides in main, alternate and IFPT datasets.

Table S2. Five-fold cross validation of ACP-BC on imbalanced datasets

	Acc(%)	Sen(%)	Spc(%)	MCC	AUC	AUPR
1	88.59	67.52	92.88	0.60	0.90	0.72
2	87.84	62.65	93.32	0.58	0.89	0.71
3	88.37	61.76	94.33	0.59	0.89	0.70
4	89.99	71.51	94.19	0.66	0.91	0.73
5	87.84	59.44	94.66	0.59	0.88	0.67

Table S3. Five-fold cross validation of ACP-check on imbalanced datasets

	Acc(%)	Sen(%)	Spc(%)	MCC	AUC	AUPR
1	83.75	47.13	91.19	0.40	0.76	0.41
2	86.01	36.74	96.72	0.44	0.76	0.51
3	84.28	49.41	92.09	0.44	0.78	0.48
4	72.77	51.74	77.54	0.25	0.68	0.31
5	83.21	61.10	88.52	0.48	0.80	0.50

Table S4. P-values from paired T-test comparing five-fold cross validation of ACP-DRL with ACP-BC and ACP-check

	Acc	Sen	Spc	MCC	AUC	AUPR
with ACP-BC	0.05084	0.56659	0.01619	0.17003	0.01449	0.00236
with ACP-check	0.02722	0.03709	0.12003	0.00341	0.00262	0.00096
